# Impact of treating iron deficiency, diagnosed according to hepcidin quantification, on outcomes after a prolonged ICU stay compared to standard care: a multicenter, randomized, single-blinded trial

**DOI:** 10.1186/s13054-020-03430-3

**Published:** 2021-02-15

**Authors:** Sigismond Lasocki, Pierre Asfar, Samir Jaber, Martine Ferrandiere, Thomas Kerforne, Karim Asehnoune, Philippe Montravers, Philippe Seguin, Katell Peoc’h, Soizic Gergaud, Nicolas Nagot, Thibaud Lefebvre, Sylvain Lehmann, the Hepcidane study group, Sigismond Lasocki, Pierre Asfar, Samir Jaber, Martine Ferrandiere, Thomas Kerforne, Karim Asehnoune, Philippe Montravers, Philippe Seguin, Katell Peoc’h, Soizic Gergaud, Nicolas Nagot, Thibaud Lefebvre, Sylvain Lehmann, François Beloncle, Alain Mercat, Thomas Gaillard, Maxime Leger, Emmanuel Rineau, Cyril Sargentini, Claire Geneve, Herve Puy, Grégoire Mercier, Gregory Marin, Constance Delaby, Christophe Hirtz, Gerald Chanques, Antoine Roquilly, Matthieu Boisson, Claire Dahyot-Fizelier, Olivier Mimoz, Sonia Isslame, Yoann Launey, Mathilde Barbaz

**Affiliations:** 1 Département Anesthésie Réanimation, CHU Angers, Université D’Angers, 4 rue Larrey, 49933 Angers Cedex 9, France; 2 Département Médecine Intensive Réanimation, CHU Angers, Université D’Angers, Angers, France; 3 Département Anesthésie Réanimation, Université de Montpellier, Montpellier, France; 4 Département Anesthésie Réanimation, CHU de Tours, Université de Tours, Tours, France; 5 Service D’anesthésie-réanimation, CHU de Poitiers, Université de Poitiers, Poitiers, France; 6 Département Anesthésie Réanimation, CHU de Nantes, Université de Nantes, Nantes, France; 7 Département Anesthésie Réanimation, APHP, HUPNSV, CHU Bichat, Université Paris Diderot Sorbonne, Paris, France; 8 Département Anesthésie Réanimation, CHU de Rennes, Université de Rennes, Rennes, France; 9 INSERM U1149, UFR de Médecine Bichat, Centre de Recherche Sur L’Inflammation, Université de Paris, Paris, France; 10 APHP Nord Hôpital Universitaire Louis Mourier, Assistance Publique des Hôpitaux de Paris, Colombes, France; 11 Laboratoire D’Excellence GR-Ex Ou Laboratory of Excellence GR-Ex, Paris, France; 12 Département D’information médicale, CHU Montpellier, Université de Montpellier, Montpellier, France; 13 Laboratoire de Biochimie Protéomique Clinique Et IRMB INSERM, CHU de Montpellier, Université de Montpellier, Montpellier, France

**Keywords:** Critically ill, Anemia, Iron deficiency, Iron (treatment), Hepcidin, Mortality, Length of stay, Erythropoietin

## Abstract

**Background:**

Anemia is a significant problem in patients on ICU. Its commonest cause, iron deficiency (ID), is difficult to diagnose in the context of inflammation. Hepcidin is a new marker of ID. We aimed to assess whether hepcidin levels would accurately guide treatment of ID in critically ill anemic patients after a prolonged ICU stay and affect the post-ICU outcomes.

**Methods:**

In a controlled, single-blinded, multicenter study, anemic (WHO definition) critically ill patients with an ICU stay ≥ 5 days were randomized when discharge was expected to either intervention by hepcidin treatment protocol or control. In the intervention arm, patients were treated with intravenous iron (1 g of ferric carboxymaltose) when hepcidin was < 20 μg/l and with intravenous iron and erythropoietin for 20 ≤ hepcidin < 41 μg/l. Control patients were treated according to standard care (hepcidin quantification remained blinded). Primary endpoint was the number of days spent in hospital 90 days after ICU discharge (post-ICU LOS). Secondary endpoints were day 15 anemia, day 30 fatigue, day 90 mortality and 1-year survival.

**Results:**

Of 405 randomized patients, 399 were analyzed (201 in intervention and 198 in control arm). A total of 220 patients (55%) had ID at discharge (i.e., a hepcidin < 41 μg/l). Primary endpoint was not different (medians (IQR) post-ICU LOS 33(13;90) vs. 33(11;90) days for intervention and control, respectively, median difference − 1(− 3;1) days, *p* = 0.78). D90 mortality was significantly lower in intervention arm (16(8%) vs 33(16.6%) deaths, absolute risk difference − 8.7 (− 15.1 to − 2.3)%, *p* = 0.008, OR 95% IC, 0.46, 0.22–0.94, *p* = 0.035), and one-year survival was improved (*p* = 0.04).

**Conclusion:**

Treatment of ID diagnosed according to hepcidin levels did not reduce the post-ICU LOS, but was associated with a significant reduction in D90 mortality and with improved 1-year survival in critically ill patients about to be discharged after a prolonged stay.

***Trial registration*:**

www.clinicaltrial.gov NCT02276690 (October 28, 2014; retrospectively registered)

## Background

Anemia is common in critically ill patients with more than 60% of them being anemic on intensive care unit (ICU) admission and more than 80% at discharge [1–3]. The two main factors contributing to this anemia are inflammation and iron deficiency (ID) [4]. ID has been found in up to 40% of critically ill patients on ICU admission [5–7]. Because these patients have important blood losses during their stay (due to repeated blood sampling, occult bleedings, surgeries, extracorporeal circuits, etc.) [8], which can exacerbate ID, higher prevalence of ID is expected on ICU discharge. Consequently, iron deficiency is the underlying etiology for anemia in ICU patients. ID at discharge from ICU has been associated with patient fatigue [9]. Indeed, iron is predominantly used for hemoglobin synthesis but also essential to cellular function and energy production processes in all human/living cells (mainly for ATP production in the mitochondria). A shortage of iron therefore impacts many aspects of cellular function including aerobic metabolism resulting in fatigue and muscle weakness, even in the absence of anemia [10]. Correcting ID improves patients’ resistance to exercise and decreases their fatigue [11, 12]. One may thus speculate that treating ID in critically ill patients may shorten their rehabilitation and thus their hospital stay post-ICU.

The problem is diagnosing ID in the presence of inflammation as laboratory markers of ferritin or transferrin saturation are often inaccurate and ferritin is elevated as part of the acute phase response [13]. In the last decades, the understanding of iron metabolism has been markedly improved by the discovery of its master regulator, hepcidin [4]. A low hepcidin level has been shown to indicate ID in critically ill patients [4, 5, 14, 15]. Data on hepcidin analysis in ICU suggest that 37% of patients have ID on ICU discharge and this group of patients had worse outcomes at 1 year, a low hepcidin being an independent predictor of 1-year post-ICU mortality [15].

We hypothesized that using hepcidin quantification to identify and treat ID in anemic patients about to be discharged after a prolonged ICU stay will reduce the length of their post-ICU hospital stay as compared to standard of care.

## Methods

### Study design

We conducted a randomized, controlled, single-blinded, multicenter (*n* = 8, French university hospital ICUs) trial. The protocol has been published elsewhere [16].

### Patients

Adult patients with anemia (according to the World Health Organization definitions, for men: hemoglobin (Hb) < 13 g/dL and for women: Hb < 12 g/dL) hospitalized in the ICU for an expected duration of ≥ 5 days were included if about to be discharged alive. Exclusions included those with known iron metabolism pathology (such as hemochromatosis), chronic anemia (defined as an Hb ≤ 10 g/dL for more than three months), current chemotherapy, organ transplant, expected survival time < 28 days post-discharge, pregnancy, inability to answer a questionnaire for neurological reasons or because of language difficulties (non-French speakers), or in case of contra-indications to intravenous iron and/or erythropoietin (EPO).

### Randomization and blinding

Patients were included when discharge from ICU was expected (and if their ICU-stay was expected to last ≥ 5 days) and allocated at random to two arms: the intervention and the control arm. Randomization was minimized on study site, age (< vs ≥ 65 years old), severity of the anemia (Hb < 8 g/dL and/or transfusion during the previous week vs. Hb ≥ 8 g/dL and no transfusion during the previous week) and the reason for admission (trauma vs. non-trauma), based on a 1:1 ratio, using an Internet server (Capture System® Software). Blinding was achieved based on results of the hepcidin quantification, which was only available online in the eCRF (and by email sent to the ICU-physician recruiting the patient) in the hepcidin arm. They did not appear in patient’s file. The patient and the non-ICU physicians remained blinded to these results.

### Trial interventions

Due to logistical reasons, mass spectrometry hepcidin quantification was only available on Thursdays (all carried out centrally at the same laboratory by TL and KP). Thus, patients about to be discharged in the following days were screened to be included between Mondays and Wednesdays. Once included, a blood sample was collected on Wednesdays to be shipped on time to the central laboratory for hepcidin quantification using our validated mass spectrometry method [17]. The investigators were informed about the results electronically (by email, on the same day, for patients in the intervention arm). For patients in the control arm, blood samples were drawn on inclusion and stored at − 80 °C to perform hepcidin quantification at the end of the study, when all the samples were available.

In the intervention arm, absolute ID was defined as a hepcidin level of < 20 μg/L (as it corresponds to the hepcidin < 130 μg/L cutoff value we observed using an Elisa method [5]) and functional ID as a 20 ≤ hepcidin < 41 μg/L (we arbitrary choose this upper limit as it is twofold the cutoff value for absolute ID). Intravenous iron was used for absolute ID treatment, using ferric carboxymaltose (1 g of iron over 15 min, according to product characteristics, see [16] for details). Functional iron deficiency had to be treated using ferric carboxymaltose (also 1 g intravenously) and one injection of erythropoietin (EPO, epoetin alpha (Eprex™, Janssen, France) 40.000 UI sub-cutaneously). Indeed, we have previously demonstrated that EPO was able to repress hepcidin synthesis, allowing iron mobilization from stores [18]. EPO injection was repeated weekly, if the patient remained anemic and in the ICU. In the control arm, ID diagnosis and treatment was left at the physician’s discretion.

Three visits were scheduled after ICU discharge: on day 15 with a blood sample to assess iron profile, hepcidin quantification and Hb concentration (for patients still hospitalized), on day 30 to assess fatigue (using the multidimensional fatigue inventory 20 (MFI-20) score [19] and a numerical scale for general fatigue (ranging from 0 = no fatigue to 10 = exhausted)) and on day 90, to assess the vital status and the history of all hospital stays post-ICU. We obtained the vital status at 90 days and at one-year of all the patients by interviewing the relevant local authorities of the patients places of residence.

### Outcomes

The primary endpoint was the length of stay (LOS) after leaving the ICU (i.e., D0 = day of ICU discharge for the first time in case the patients were readmitted to the ICU), calculated as the number of hospitalization calendar days between discharge from the ICU and D90. Secondary outcomes were prevalence of ID and mean Hb concentrations on D15, prevalence of fatigue on D30 (based on the scores obtained using the MFI-20 questionnaire for the four different dimensions of fatigue and on a general fatigue assessment using a numerical scale graded from 0 to 10), the percentage of patients alive and at home on D90, the mortality rates on D90 and the one-year survival after ICU discharge.

### Statistical analysis

All results are expressed as mean ± SD or median (IQR) for continuous variables, depending on their distribution or n(%) for categorical variables. The statistical analysis followed a prespecified plan [16]. First, data were analyzed using the intention-to-treat (ITT) principle according to their study arm, taking all the patients randomized and fulfilling the inclusion/exclusion criteria. Then, a pre-specified subgroup analysis was performed, to assess the effect of treatment (iron ± EPO) in patients with ID (i.e., in patients having hepcidin concentrations < 41 µg/L). For these subgroups’ analyses, patients with ID treated in the intervention arm were compared to patients with ID (defined according to hepcidin quantification) not treated in the control arm.

For the primary endpoint, since the distribution of patients’ LOS is not usually normal, nonparametric tests were used (i.e., Mann–Whitney test) in order to compare the number of days of post-ICU hospitalization between the two experimental arms. For patients with missing data, LOS was arbitrary set at 90 days. Sample size calculation was based on the hypothesis that LOS values obtained in the intervention arm will be shorter than in the control arm in 60% of the pairs compared (taking into account that at least 50% of the patients will have ID in both groups and that treatment of ID in the intervention arm will improve LOS); with a 5% alpha risk and a 90% power, the total number of patients required for this study was 400 [20]. Since the study covers a relatively short period and the duration of patients’ hospitalization is an easy to obtain variable, we did not expect any loss of follow up to occur. However, the number of patients was increased to 405 to compensate for patients included but not leaving the ICU alive.

To account for possible cofounders, we used a linear regression and adjusted this analysis according to the principal factors imbalanced between the two arms and expected to be associated with post-ICU LOS: diabetes and McCabe score [21]. In subgroup analysis (ID treated vs not treated), linear regression analysis was performed to adjust the analysis according to the centers for the post-ICU LOS analysis. The secondary outcomes were compared between the two arms by Wilcoxon–Mann–Whitney or Chi-square tests. A logistic regression was executed to analyze the impact of study arm on mortality at 90 days, after adjusting on confounding variables. Univariate analyses were first carried out, taking into account variable of interest with regard to mortality. Then, the variables with a p value lower than 0.15 were considered for a multivariate logistic model. The variables with a p value lower than 0.05 in the multivariate model after a stepwise selection of variables were considered statistically significant. Finally, we compared the one-year survival after ICU discharge alive using Kaplan–Meier curves. For all tests, *p* < 0.05 was considered statistically significant. All statistical analyses were performed using SAS V9.2 (SAS Institute Inc, Cary, NC, USA).

## Results

### Baseline characteristics

**Fig. 1:**
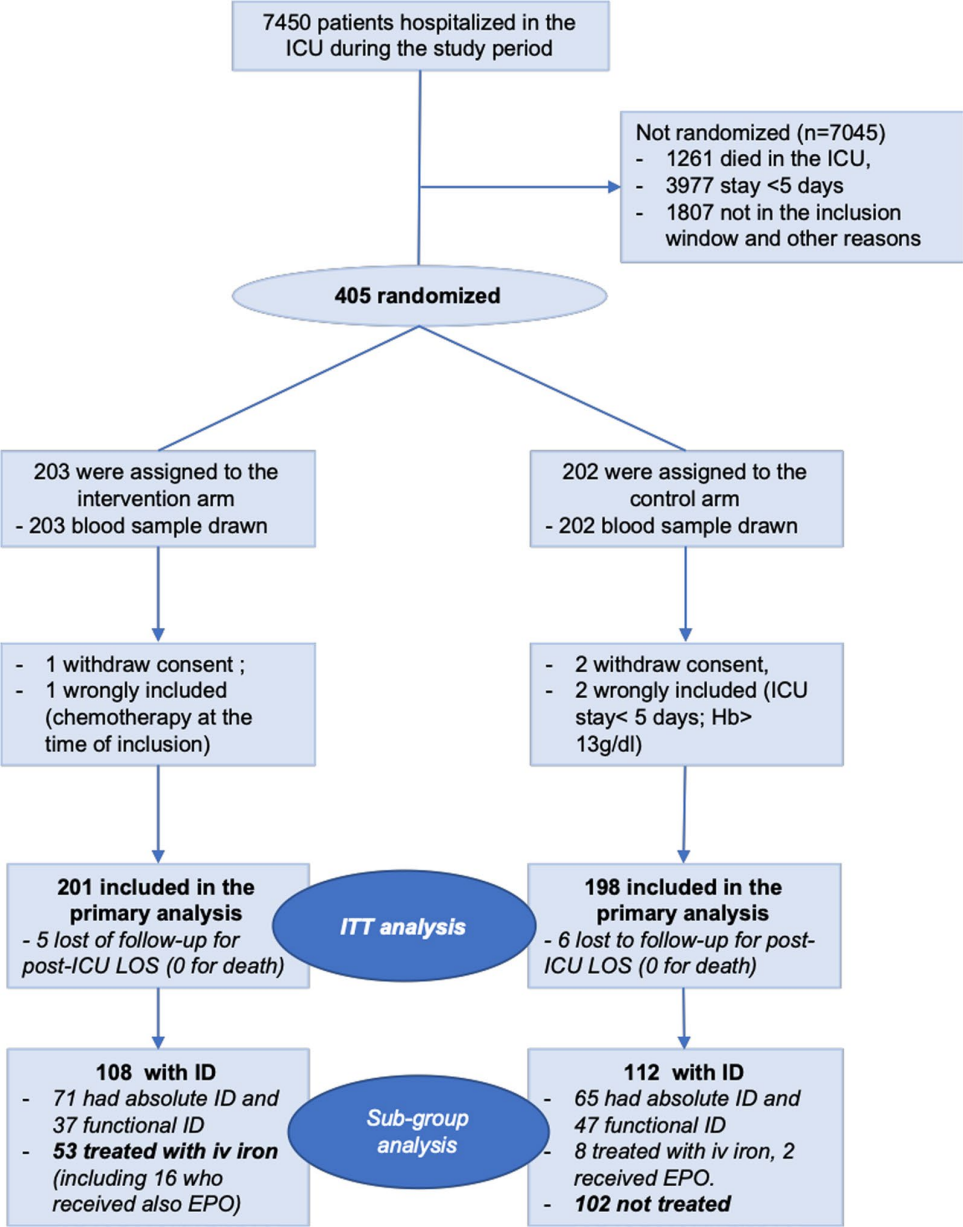
Screening, randomization, and follow-up of patients in the hepcidin trial. *ICU* intensive care unit, *ID* iron deficiency, *Hb* hemoglobin, *Absolute ID* absolute iron deficiency was defined as an hepcidin < 20 µg/L, *Functional ID* functional iron deficiency was defined as 20 ≤ hepcidin < 41 µg/L, *ITT analysis* intention-to-treat analysis. A subgroup analysis was scheduled and compared the patients with ID treated in the intervention arm to patients with ID not treated in the control arm

Between August 1, 2014 and June 30, 2016, 405 patients were included and randomized in the eight participating centers, among them 399 patients were analyzed (201 in the intervention group and 198 in the control group, see Fig. 1 for flowchart) in ITT analysis. Additional file 1: Table S1 detailed the number of inclusions at each center. The median (IQR) age was 65(55; 74) years, with 270 (68%) men. Two hundred forty-five (61%) patients had a surgery prior to ICU hospitalization. The median Simplified Acute Physiology Score II (SAPS II) on admission was 40(28; 53), 318 (80%) patients were ventilated, and 262 (66%) received catecholamine. Baseline characteristics of the patients are depicted in Table 1.

**Table 1:** Patients characteristics according to the study group

	Intervention (*n* = 201)	Control (*n* = 198)	*p*
Age (years)	63.4 ± 14.8	63.1 ± 14.3	0.77
Women	60 (31.1)	65 (33.5)	0.67
BMI (kg/m^2^)	27.7 (23.4; 32.8)	27.1 (24.1; 30.8)	0.81
At least one chronic disease	160 (79.6)	157 (79.3)	0.94
Diabetes	42 (26.3)	60 (38.2)	0.023
Cirrhosis	12 (7.5)	18 (11.6)	0.21
Heart failure	13 (8.2)	14 (8.9)	0.79
Arterial hypertension	106 (66.3)	93 (59.6)	0.22
Coronary artery disease	26 (16.3)	18 (11.6)	0.23
Chronic renal failure	13 (8.1)	13 (8.3)	0.95
COPD	23 (14.4)	21 (13.5)	0.81
*Mc CABE score*			0.21
Non-fatal	140 (71.8)	143 (74.1)	
Ultimately fatal (1–4 years)	47 (24.1)	36 (18.7)	
Rapidly fatal (< 1 year)	8 (4.1)	14 (7.3)	
*ICU admission*			
Recent surgical history	122 (60.7)	123 (62.1)	0.77
Sepsis on admission	72 (35.8)	75 (37.9)	0.67
Significant bleeding on admission	35 (17.4)	37 (18.7)	0.74
Transfusion before inclusion*	92 (45.8)	90 (44.5)	0.95
SAPS II	38 (29; 51)	41 (27; 55)	0.49
SOFA	6 (4; 9)	7 (4; 10)	0.14
Hb (g/dL)	11.1 ± 2.2	11.2 ± 2.7	0.98
*Organ support during ICU stay*			
ICU LOS (days)	13 (7; 22)	12 (7; 20)	0.89
Mechanical ventilation	158 (78.6)	160 (80.8)	0.58
Duration of MV (days)	4 (2; 12)	5 (2; 12)	0.86
Renal support	31 (15.4)	26 (13.1)	0.51
Duration of support (days)	7 (2; 14)	4 (2; 13)	0.34
Catecholamine	129 (64.2)	133 (67.2)	0.53
Duration of catecholamine (days)	3 (2; 5)	3 (2; 5)	0.68
*ICU discharge blood tests*			
Hb (g/dL)	10.0 (9.1; 10.8)	9.7 (8.8; 10.7)	0.12
CRP (mg/L)	66.0 (39.0; 102.9)	69.0 (34.0; 128.0)	0.82
Ferritin (µg/L)	710 (363; 1201)	584 (286; 903)	0.16
TSAT (%)	14 (10; 22)	14 (12; 19)	0.84
Hepcidin (µg/L)	31.10 (13.35; 56.25)	33.70 (13.55; 65.45)	0.67
Absolute ID (*n*)	71 (35.3)	65 (32.8)	0.47
Functional ID (*n*)	37 (18.4)	47 (23.7)	0.29

Data are expressed as mean ± SD, median(Q1;Q3) or *n*(%)

*BMI* Body Mass Index, *COPD* chronic obstructive pulmonary disease, *ICU* intensive care unit, *SAPS II* Simplified Acute Physiology Score II, *SOFA* Simplified Organ Failure Assessment, *Hb* hemoglobin, *LOS* length of stay, *MV* mechanical ventilation, *CRP C* reactive protein, TSAT transferrin saturation. *Absolute ID* Absolute iron deficiency was defined as an hepcidin < 20 µg/L; *Functional ID* functional iron deficiency was defined as 20 ≤ hepcidin < 41 µg/L

^*^Transfusion before inclusion is defined as having received a blood transfusion during the week before inclusion

Overall, 220 (55%) patients had ID on inclusion, with 136 (34%) having an absolute ID (hepcidin < 20 µg/L) and 84 (21%) having a functional ID (20 ≤ hepcidin < 41 µg/L). In the intervention arm, 71 (35%) patients had absolute ID, but 37 (52%) of them were not treated and 3 (4%) received EPO with iron; 37 (18%) had a functional ID, but 18 (49%) were not treated and 6 (16%) received only iron (without EPO). The median dose of iron (ferric carboxymaltose) received was 1000 (1000; 1000) mg per treated patients, received in median 10 (7; 21) days after ICU admission. The median number of EPO injections was 1 (1; 2), with 13 (68%) patients who received 1 and 6 (32%) 2 or more injections. Thus, 53 (49%) patients with ID were treated. In the control group, 11 (5%) patients received iron (median dose 800 (300; 1500) mg, received in median 9 (6; 15) days after ICU admission) and 2 (1%) received EPO (respectively, 1 and 4 injections). According to the hepcidin determination in the control group, 65 (33%) had absolute ID and 47 (24%) had a functional ID. Among them, 102 (91%) were not treated (see Fig. 1).

### Primary outcome

In ITT analysis, the length of hospital stay after ICU was not different between the 2 study arms (33 (13; 90) vs 33(11; 90) days for intervention and control, respectively, median difference − 1 day, 95% CI, − 3 to 1, *p* = 0.78), even after adjustment for diabetes and MacCabe score (*p* = 0.96).

### Secondary outcomes and subgroup analysis

**Fig. 2:**
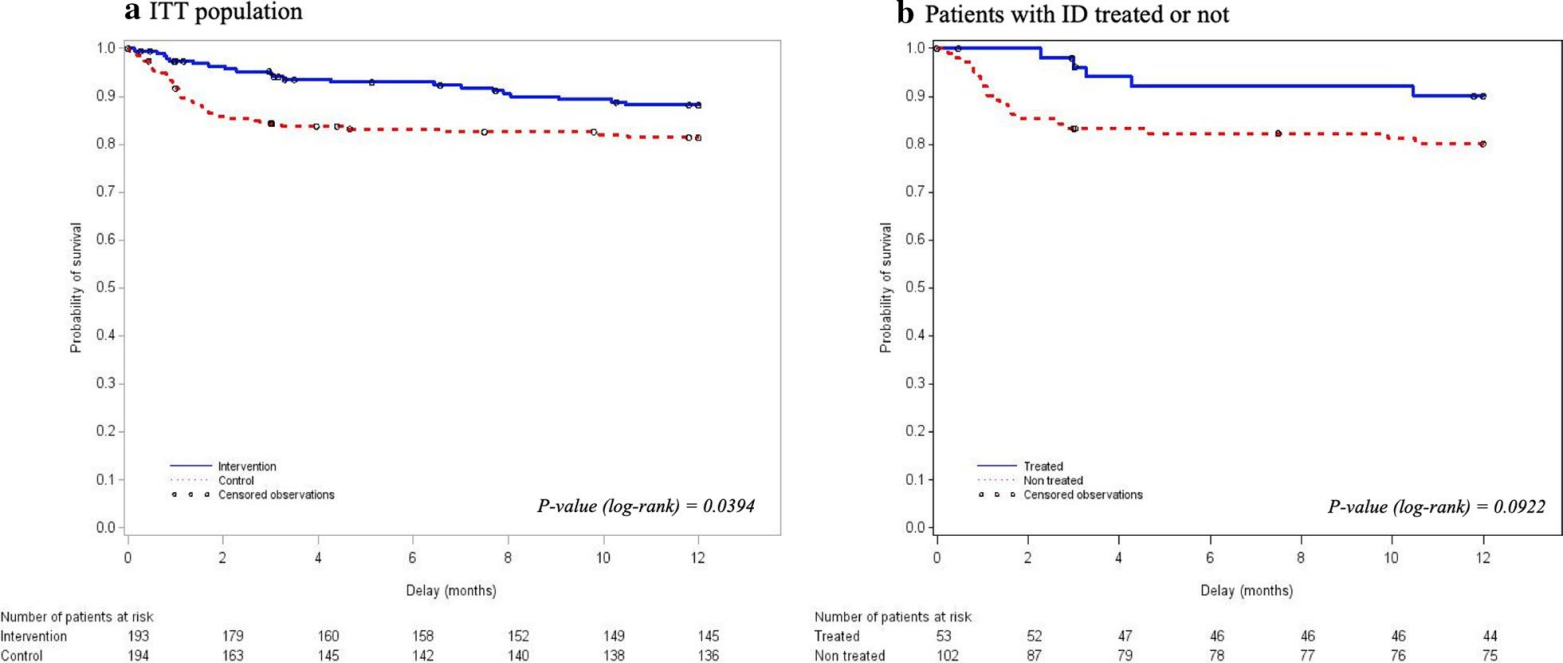
Kaplan–Meier survival curves (till D360 after ICU discharge alive). **a** Intention-to-treat analysis (all population). **b** Scheduled subgroup analysis (patients with hepcidin < 41 µg/L treated in the intervention arm and not treated in the control arm)

In the ITT analysis, there was no difference for any of the secondary endpoints, except for lower D90 mortality rate in the intervention arm (16 (8%) vs 33 (16.6%) deaths, absolute risk difference − 8.7%; 95% CI − 15.1 to − 2.3, *p* = 0.008) (Table 2). Twelve patients died before ICU discharge (8 in intervention and 4 in control arm). We conducted a logistic regression analysis and found that study arm (OR 0.46, 95% CI 0.22–0.94, *p* = 0.035), age (OR 1.07, 95% CI 1.02–1.12 for each year, *p* < 0.001) and duration of mechanical ventilation (OR 1.05, 95% CI 1.02–1.09 for each day, *p* < 0.001) were predictive of D90 mortality (see Additional file 1: Table S2 for details). Finally, the one-year survival after ICU discharge alive was also significantly improved in the intervention arm (Fig. 2, Panel A).

**Table 2:** Primary and secondary outcomes (ITT analysis)

	*n*	Intervention (*n* = 201)	*n*	Control (*n* = 198)	*P*
*Primary endpoint*					
Post-ICU LOS (days)	201	33 (13; 90)	198	33 (11; 90)	*0.78*
*Secondary outcomes*					
Day 15 Hb (g/dL)	127	10.3 ± 1.7	119	10.30 ± 1.5	*0.81*
Day 15 hepcidin (µg/L)	69	34.6 (17.4; 53.7)	68	27.7 (11.9; 62.7)	*0.63*
Day 30 fatigue (scale 1–10)	120	5 (3; 6)	114	5 (3; 7)	*0.44*
Day 30 MFI-20,	126		120		
General fatigue (score 9–45)	126	27 (18; 31)	120	24*.5 (18; 30)*	*0.41*
Mental fatigue (score 6–30)	126	24 (20; 28)	120	26 (21; 29)	*0.26*
Reduced activity (score 3–15)	126	8 (6; 10)	120	8 (6; 11)	*0.66*
Reduced motivation (score 2–10)	126	8 (6; 10)	120	8 (6; 10)	*0.72*
Deaths at day 90	201	16 (8.0)	198	33 (16.67)	0.008

Data are expressed as mean ± SD, median(Q1; Q3) or n(%)

Italics were used to indicate sub-headings in the tables and *p*-values

Somme data are missing (i.e., Hb concentrations at D15, fatigue assessment at D30), in that cases the number of available data is indicated. *ITT* intention to treat, *ICU* intensive care unit, *LOS* length of stay, *Hb* hemoglobin, *MFI-20* multidimensional fatigue inventory

In the prespecified subgroup analysis, we compared these outcomes in patients with ID: taking into account the 53 patients with ID (hepcidin < 41 µg/L) who have been treated in the intervention arm and the 102 patients with ID (hepcidin < 41 µg/L) not treated in the control arm. The two groups were comparable with regard to main patient’s characteristics (see Additional file 1: Table S3). We found no difference in the primary outcome (post-ICU LOS 42 (16; 90) vs. 29 (10; 90) days, median difference 4.5, − 1 to 10 days, *p* = 0.37) or in main secondary outcomes. This absence of difference persists after adjustment on centers (data not shown). Only the D15 hepcidin concentration was higher in the treated patients, suggesting higher iron stores, without significant differences in hemoglobin levels (Table 3). In this subgroup analysis, D90 mortality was dramatically reduced in ID treated patients (2 (3.8%) vs 17 (16.7%) deaths, absolute risk difference − 12.9%; 95% CI, − 21.7 to − 4.0, *p* = 0.002). Interestingly, the D90 mortality of patients without ID was similar in both study arms (8 (9.6%) vs 10 (13.3%) deaths, absolute risk difference − 3.69, − 13.675 to 6.28, *p* = 0.47 in non-ID patients for, respectively, the intervention (*n* = 83) and control (*n* = 75) arms). The 1-year survival after ICU discharge alive was improved in this subgroup, without reaching statistical significance (Fig. 2, panel B). This analysis was also conducted per protocol, comparing the 47 patients with ID treated according to the protocol in intervention arm (not taking into account the 6 patients with functional ID who did not received EPO) to the 102 patients with ID not treated in control arm and found the same results (see Additional file 1: Table S4 and Figure S1).

**Table 3:** Effect of iron deficiency treatment (scheduled subgroup analysis)

	*n*	Patients with ID treated in hepcidin arm (*n* = 53)	*n*	Patients with ID not treated in control arm (*n* = 102)	*p*
*Primary outcome*					
Post-ICU LOS (days)	53	42 (16; 90)	102	29 (11; 90)	*0.37*
*Secondary outcomes*					
Number of days alive at home at day 90	53	50 (0; 76)	102	61 (0; 82)	*0.21*
*Day 15 visit*					
Day 15 Hb (g/dL)	40	10.7 ± 1.6	59	10.4 ± 1.4	*0.24*
Day 15 hepcidin (µg/L)	19	35.7 (23.0; 53.7)	31	18.0 (7.6; 44.0)	*0.04*
Day 30 visit					
Fatigue (scale 1–10)	39	5.0 (3.0; 6.0)	61	6.0 (3.0; 7.0)	*0.13*
MFI-20					
General fatigue (score 9–45)	40	28 (22; 31)	63	26 (18; 30)	*0.33*
Mental fatigue (score 6–30)	40	25 (20; 28)	63	25 (20; 28)	*0.75*
Reduced activity (score 3–15)	40	8 (6;10)	63	8 (6;11)	*0.66*
Reduced motivation (score 2–10)	40	8.(6;10)	63	8 (6; 10)	*0.24*
*Mortality*					
Death at day 90	53	2 (3.8)	102	17 (16.7)	*0.02*

Data are expressed as mean ± SD, median(Q1;Q3) or n(%). We compared the outcomes of patient with iron deficiency (ID, defined as an hepcidin concentration < 41 µg/L) treated in the hepcidin arm to the patients with ID not treated in the control arm

Italics were used to indicate sub-headings in the tables and *p*-values

*ICU* intensive care unit, *LOS* length of stay, *MFI-20* multidimensional fatigue inventory, *Hb* hemoglobin

^*^ Absolute risk difference

## Discussion

In this randomized controlled trial, including anemic patients about to be discharged from ICU after a prolonged ICU stay, a strategy of diagnosing and treating ID according to hepcidin quantification allows for the frequent identification of ID but did not reduce the post-ICU LOS compared to standard of care. However, this strategy reduced the D90 post-ICU mortality by 50% and improved the 1-year survival.

This trial has several strengths. First, contrary to previous trials, evaluating the benefit of iron in critically ill, aimed at reducing blood transfusion [22–24], we chose to evaluate the benefit of treating iron deficiency (rather than giving iron to all patients to treat anemia). Indeed, giving intravenous iron to patients without ID may increase the risk of iron side-effects and of iron overload, whereas giving iron in critically ill patients with ID does not expose to an increased risk of oxidative stress [25]. Second, we used a new biomarker to identify ID, hepcidin [4, 5, 14, 15], because standard laboratory tests are difficult to interpret in the presence of inflammation [13, 26, 27]. We used a validated mass spectrometry method [17], which is relatively cheap and easy to obtain. These tests were developed years ago [28, 29] and will probably be standardized soon [30]. Third, we focus on the post-ICU period, since rehabilitation and post-ICU survival are now recognized as important outcomes, since post-ICU quality of life is frequently poor and mortality rates high [31]. Fourth, although double blinding was impossible, the patient and the post-ICU physicians remained blinded to the study arm. At last, we evaluated a mix-ICU population, including medical and surgical patients, increasing the external validity of our results.

Our results demonstrate that iron deficiency may be recognized in a large proportion of critically ill patients (more than 50%). This is consistent with the high proportion of ID observed on ICU admission (between 20 and 40% using different parameters) [5–7], and with the proportion of ID (defined as a low hepcidin concentration), we and others reported [14, 15]. These prevalence are much higher than the ones observed using standard laboratory tests (less than 10%) [9], confirming the interest of hepcidin quantification as a new ID diagnostic method. However, as with any diagnostic tool, one should bear in mind that cutoff values should be analyzed according to the clinical context and that the lower the hepcidin, the greater the likelihood of iron deficiency. For example, in healthy blood donor a hepcidin threshold of < 10 µg/L is indicating of ID [32].

There was no difference in post-ICU LOS between the 2 study arms. It may be that fatigue is not the only (or the main) determinant of post-ICU LOS and/or that treating ID was not sufficient to improve fatigue. Indeed hospital discharge is also dependent on many logistical and organizational factors not directly linked to the patient’s condition. It is also possible that the dose of iron we used was not sufficient. Indeed, following the product characteristics patients weighing more than 70 kg should often have received a second injection of iron, but this was never done since patients were discharged from the ICU at that time. The prevalence of ID and the Hb concentration on D15 were not different in treated and not treated patients. Even if the treatment was efficient to increase hepcidin on D15, indicating an increase in iron stores, a large proportion of patients remained iron deficient according to our definition. It is also possible that Hb concentrations were higher later but not measured. These analyses concern few patients; one should thus remain cautious regarding these results.

In our study, we observed an important reduction (around 50%) of D90 mortality rate in the intervention arm in both *intention-to-treat* and *sub-group* analyses as well as an improved one-year survival in patients discharged alive from ICU. This is consistent with the observed increase in one-year mortality reported in critically ill patients with low hepcidin at discharge from ICU [14, 15], and with the results of a recent study in hemodialysis patients, showing that treating ID with higher doses of iron reduces the number of hospitalization episodes (for heart failure) [33] and with improved outcome observed in ID treatment of heart failure patients [34]. It is thus largely plausible that treating ID improved post-ICU survival. In addition, EPO treatment has also been shown to reduce mortality in critically ill patients and may have contributed to the lower mortality rate we observed [35, 36]. It is now recommended (low grade recommendation) by the French societies of critical care to treat anemia with erythropoietin in ICU [37]. Importantly, we did not observe any side-effects of ID treatment (neither related to IV iron nor to EPO), so that the benefit-risk balance seems largely positive [38].

This trial has several limitations. First, we observed a relatively high rate of protocol violation (i.e., patients with ID not treated in the intervention arm). This is mainly due to the logistic constraints of the study. Indeed, because patients were screened and included exclusively between Mondays and Wednesdays, some patients had left the ICU before the results of the hepcidin dosage were available, and were not followed after ICU discharge (non-ICU physicians had no access to hepcidin dosage). We thus cannot exclude a lack of power of our study to detect a difference in post-ICU LOS, but this is unlikely in regard of our results. Second, we have a lot of missing data for the D15 blood samples, so that we cannot evaluate the effect of the intervention on D15 ID and anemia prevalence. Third, we also lack measurement of D30 fatigue. But we have no missing data for the D90 and D360 mortality, which are much more important outcomes to a clinical point of view. Fourth, we do not have the cause of mortality. At last, we did not conduct the scheduled medico-economic analysis, since we did not observe any reduction in post-ICU LOS, the main driver for cost effectiveness of the intervention.

## Conclusion

Treating iron deficiency, diagnosed according to hepcidin quantification, on ICU discharge did not reduce the post-ICU LOS, but was associated with a significant reduction in D90 mortality and with improved 1-year survival, in anemic critically ill patients about to be discharged after a prolonged ICU stay.

## Supplementary Information

**Additional file 1.** **eTable 1:** Description of centers and number of inclusions by center; **eTable 2:** univariate and multivariate analysis for day 90 Mortality; **eTable 3:** Patients characteristics in sub-group analysis, comparing ID patients treated in intervention arm to ID patients not treated in control arm; **eTable 4:** per protocol analysis of primary and secondary outcomes and **eFigure 1:** Kaplan-Meier survival curves (till D90) in patients with hepcidin <41 μg/L treated in the intervention arm according to the study protocol and not treated in the control arm.

## Data Availability

The corresponding author (SL) had full access to all the study data. The corresponding author (SL) takes responsibility for the integrity of the data and the accuracy of the data analysis. The datasets used and/or analyzed during the current study are available from the corresponding author on reasonable request.

## Abbreviations

EPO: Erythropoietin
Hb: Hemoglobin
ID: Iron deficiency
ICU: Intensive care unit
ITT: Intention to treat
LOS: Length of stay
MFI 20: Multidimensional fatigue inventory 20
SAPS II: Simplified Acute Physiology Score II
