# Supplementary material for: Impact of treating iron deficiency, diagnosed according to hepcidin quantification, on outcomes after a prolonged ICU stay compared to standard care: a multicenter, randomized, single-blinded trial
Source: Crit Care. 2021 Feb 15;25:62. doi: 10.1186/s13054-020-03430-3 (PMC7885380; doi:10.1186/s13054-020-03430-3)
Supplement: Supplementary file 1 — Additional file 1. eTable 1: Description of centers and number of inclusions by center; eTable 2: univariate and multivariate analysis for day 90 Mortality; eTable 3: Patients characteristics in sub-group analysis, comparing ID patients treated in intervention arm to ID patients not treated in control arm; eTable 4: per protocol analysis of primary and secondary outcomes and eFigure 1: Kaplan-Meier survival curves (till D90) in patients with hepcidin <41 μg/L treated in the intervention arm according to the study protocol and not treated in the control arm. [file 13054_2020_3430_MOESM1_ESM.docx]

**Additional file 1**

**Hepcidin, Iron deficiency treatment and post-ICU outcomes**

Sigismond LASOCKI et al.

**Table S1: Description of centers and number of inclusions by center**

All the centers are university-hospitals. Data are expressed as n(%) or median (Q1;Q3)

SAPSII, simplified acute physiology score.

| **Site number** | | | | | | | | | |
| --- | --- | --- | --- | --- | --- | --- | --- | --- | --- |
|  | **Total population** | **1** | **2** | **3** | **4** | **5** | **6** | **7** | **8** |
|  |  |  |  |  |  |  |  |  |  |
| **Type of ICU** |  | **Mixte CU** | **Surgical ICU** | **Surgical ICU** | **Surgical ICU** | **Surgical ICU** | **Surgical ICU** | **Surgical ICU** | **Medical ICU** |
| Number of patients included | 399 | 66 | 16 | 92 | 42 | 14 | 23 | 66 | 80 |
| - *control arm* | 198 (49.6) | 33 (50) | 7 (43.8) | 47 (51.1) | 20 (47.6) | 7 (50) | 12 (52.2) | 34 (51.5) | 38 (47.5) |
| - *Intervention arm* | 201 (50.4) | 33 (50) | 9 (56.2) | 45 (48.9) | 22 (52.4) | 7 (50) | 11 (47.8) | 32 (48.5) | 42 (52.5) |
| SAPSII Score | 40 (28 ; 53) | 31 (24 ; 40) | 36 (28 ; 53) | 43 (32; 57) | 38 (28 ; 50) | 41 (30 ; 57) | 39 (26 ; 60) | 46 (31 ; 55) | 41 (33 ; 58) |
| Type of admission : surgical | 245 (61.4) | 34 (51.5) | 14 (87.5) | 82 (89.1) | 29 (69.1) | 12 (85.7) | 19 (82.6) | 51 (77.3) | 4 (5) |
| Type of admission : medical | 154 (38.6) | 32 (48.5) | 2 (12.5) | 10 (10.9) | 13 (30.9) | 2 (14.3) | 4 (17.4) | 15 (22.7) | 76 (95) |

**Table S2: univariate (upper table) and multivariate analysis (lower table) for day 90 Mortality**

A logistic regression was executed to analyze the impact of study arm on mortality at 90 days, after adjusting on confounding variables. Univariate analyses were first carried out, taking into account variable of interest with regard to mortality. Then, the variables with a p-value lower than 0.15 were considered for a multivariate logistic model. The variables with a p-value lower than 0.05 in the multivariate model after a stepwise selection of variables were considered statistically significant.

COPD, chronic obstructive pulmonary disease; ID, iron deficiency; BMI, body mass index; SAPSII, simplified acute physiology score; SOFA, Sequential Organ Failure Assessment; MV, mechanical ventilation; CRP, c reactive protein; ICU, intensive care unit.

| **variable** |  | **Alive at Day-90**  **(n=347)** | **Dead at Day-90**  **(n=52)** | **StatisticalTest** | **p** |
| --- | --- | --- | --- | --- | --- |
| Study Arm | Control | 164 (82.83) | 34 (17.17) | CHI2 | 0.01 |
|  | Intervention arm | 183 (91.04) | 18 (8.96) |  |  |
| Gender | Women | 112 (86.82) | 17 (13.18) | CHI2 | 0.95 |
|  | Men | 235 (87.04) | 35 (12.96) |  |  |
| At least one chronic disease | No | 78 (95.12) | 4 (4.88) | CHI2 | 0.01 |
|  | Yes | 269 (84.86) | 48 (15.14) |  |  |
| Alcohol consumption | No | 214 (84.58) | 39 (15.42) | CHI2 | 0.94 |
|  | Yes | 51 (85.00) | 9 (15.00) |  |  |
| Obesity | No | 187 (85.00) | 33 (15.00) | CHI2 | 0.89 |
|  | Yes | 81 (84.38) | 15 (15.63) |  |  |
| Diabetes | No | 186 (86.51) | 29 (13.49) | CHI2 | 0.23 |
|  | Yes | 83 (81.37) | 19 (18.63) |  |  |
| Arterial Hypertension | No | 98 (83.76) | 19 (16.24) | CHI2 | 0.69 |
|  | Yes | 170 (85.43) | 29 (14.57) |  |  |
| Coronary artery disease | No | 229 (84.19) | 43 (15.81) | CHI2 | 0.45 |
|  | Yes | 39 (88.64) | 5 (11.36) |  |  |
| Chronic Renal Failure | No | 249 (85.86) | 41 (14.14) | FISHER | 0.09 |
|  | Yes | 19 (73.08) | 7 (26.92) |  |  |
| Cirrhosis | No | 248 (87.02) | 37 (12.98) | FISHER | < 0.01 |
|  | Yes | 19 (63.33) | 11 (36.67) |  |  |
| Respiratory insufficiency | No | 253 (86.64) | 39 (13.36) | FISHER | < 0.01 |
|  | Yes | 15 (62.50) | 9 (37.50) |  |  |
| COPD | No | 233 (85.66) | 39 (14.34) | CHI2 | 0.29 |
|  | Yes | 35 (79.55) | 9 (20.45) |  |  |
| Mac CABE Score | Non-fatal | 256 (90.46) | 27 (9.54) | FISHER | 0.02 |
|  | Ultimately fatal | 66 (79.52) | 17 (20.48) |  |  |
|  | Rapidly fatal | 18 (81.82) | 4 (18.18) |  |  |
| Patient's type of admission | Surgical | 218 (88.98) | 27 (11.02) | CHI2 | 0.13 |
|  | Medical | 129 (83.77) | 25 (16.23) |  |  |
| Sepsis on admission | Non | 222 (88.10) | 30 (11.90) | CHI2 | 0.38 |
|  | Oui | 125 (85.03) | 22 (14.97) |  |  |
| Significant bleeding on admission | No | 282 (86.24) | 45 (13.76) | CHI2 | 0.36 |
|  | Yes | 65 (90.28) | 7 (9.72) |  |  |
| Trasnfusion before inclusion | No | 192 (88.48) | 25 (11.52) | CHI2 | 0.33 |
|  | Yes | 155 (85.16) | 27 (14.84) |  |  |
| Mecanical ventilation | No | 72 (88.89) | 9 (11.11) | CHI2 | 0.57 |
|  | Yes | 275 (86.48) | 43 (13.52) |  |  |
| Non invasive ventilation | No | 231 (87.83) | 32 (12.17) | CHI2 | 0.46 |
|  | Yes | 115 (85.19) | 20 (14.81) |  |  |
| Catecholamine | No | 231 (87.83) | 32 (12.17) | CHI2 | 0.13 |
|  | Yes | 115 (85.19) | 20 (14.81) |  |  |
| Iron deficiency | Absolute ID | 118 (85.51) | 20 (14.49) | FISHER | 0.90 |
|  | Functional ID | 76 (83.52) | 15 (16.48) |  |  |
|  | No ID | 145 (89.51) | 17 (10.49) |  |  |
| age | Mean (± sd) | 62.23 (± 14.70) | 71.81 (± 9.92) | WMW | < 0.01 |
| BMI | Mean (± sd) | 29.20 (± 14.32) | 28.01 (± 7.40) | WMW | 0.63 |
| SAPSII Score | Mean (± sd) | 40.14 (± 17.12) | 49.90 (± 15.98) | WMW | < 0.01 |
| SOFA total score | Median (Q1;Q3) | 6.00 (4.00 ; 9.00) | 8.00 (4.00 ; 10.00) | WMW | 0.10 |
| Hepcidine | Median (Q1;Q3) | 34.00 (14.00 ; 62.80) | 24.60 (13.10 ; 55.35) | WMW | 0.27 |
| Duration of MV (days) | Median (Q1;Q3) | 4.00 (2.00 ; 11.00) | 9.00 (3.00 ; 18.00) | WMW | < 0.01 |
| Duration of catecholamines (days) | Median (Q1;Q3) | 3.00 (2.00 ; 4.00) | 5.00 (2.00 ; 10.00) | WMW | < 0.01 |
| Hemoglobin at admission | Mean (± sd) | 11.18 (± 2.46) | 10.78 (± 2.28) | WMW | 0.36 |
| CRP at ICU admission |  | n=128 | n=18 |  |  |
| CRP at ICU admission | Median (Q1;Q3) | 87.00 (11.90 ; 204.00) | 157.50 (29.00 ; 300.70) | WMW | 0.08 |
|  | Median (min;max) | 87.00 (1.00 ; 506.30) | 157.50 (6.00 ; 428.30) |  |  |
| Hemoglobin at ICU discharge |  | n=276 | n=38 |  |  |
|  | Median (Q1;Q3) | 9.90 (8.90 ; 10.80) | 9.60 (8.30 ; 10.10) | WMW | 0.05 |
| Ferritin at ICU discharge |  | n=151 | n=16 | WMW | 0.89 |
|  | Median (Q1;Q3) | 681.00 (310.00 ; 1043.00) | 538.00 (236.00 ; 1264.00) |  |  |
| Transferrin saturation at ICU discharge |  | n=144 | n=14 |  |  |
|  | Median (Q1;Q3) | 14.00 (11.00 ; 20.00) | 19.50 (13.00 ; 26.00) | WMW | 0.08 |
| CRP at ICU discharge |  | n=178 | n=20 |  |  |
|  | Median (Q1;Q3) | 68.50 (34.80 ; 115.00) | 57.50 (29.85 ; 137.30) | WMW | 0.92 |

**Logistic regression model for Day-90 mortality:**

|  | **Odds-ratio** | **95% Confidence Interval** | | **P-value** |
| --- | --- | --- | --- | --- |
| **Study arm : Intervention vs Control** | 0.46 | 0.22 | 0.94 | 0.035 |
| **Age (per each 1-year increase)** | 1.07 | 1.04 | 1.12 | < 0.001 |
| **Duration of Mechanical ventilation (per each 1-day increase)** | 1.05 | 1.02 | 1.09 | <0.001 |

**Table S3: Patients characteristics in sub-group analysis, comparing ID patients treated in intervention arm to ID patients not treated in control arm.**

Data are expressed as mean±SD, median(Q1;Q3) or n(%).

BMI, Body Mass Index; COPD, Chronic Obstructive Pulmonary Disease; ICU, Intensive Care Unit; SAPS II, Simplified Acute Physiology Score II; SOFA, Simplified Organ Failure Assessment; Hb, Hemoglobin; LOS, Length of stay; MV, Mechanical Ventilation; CRP, C Reactive Protein; TSAT, Transferrin Saturation. Absolute ID, Absolute iron deficiency was defined as an hepcidin <20 µg/L; Functional ID, Functional iron deficiency was defined as 20≤ hepcidin <41 µg/L.

* Transfusion before inclusion is defined as having received a blood transfusion during the week before inclusion.

|  | Patients with ID treated in Hepcidin arm  (n= 53) | Patients with ID not treated in control arm  (n=102) | p |
| --- | --- | --- | --- |
| Age (yrs) | 63.1±15.86 | 63.3±14.3 | 0.99 |
| Women | 20 (37.7) | 45 (44.1) | 0.44 |
| BMI (kg/m^2^) | 27.7 (23.4 ; 33.9) | 27.4 (24.6 ; 31.2) | 0.83 |
| At least one chronic disease | 38 (71.7) | 81 (79.4) | 0.28 |
| Diabetes | 12 (22.6) | 12 (31.3) | 0.4 |
| Cirrhosis | 3 (5.6) | 13 (12.7) | 0.21 |
| Heart Failure | 4 (7.5) | 6 (5.9) | 0.73 |
| Arterial Hypertension | 23 (43.4) | 46 (45) | 0.76 |
| Coronary artery disease | 6 (11.3) | 9 (8.8) | 0.56 |
| Mc CABE score |  |  | 0.96 |
| Non-fatal | 40 (75.5) | 74 (72.7) |  |
| Ultimately fatal (1-4 years) | 10 (18.9) | 20 (19.6) |  |
| Rapidly fatal (<1 year) | 3 (5.7) | 8 (7.8) |  |
| *ICU admission* |  |  |  |
| Recent surgical history | 31 (58.5) | 62 (60.8) | 0.78 |
| Sepsis on admission | 18 (33.9) | 38 (37.2) | 0.73 |
| Significant bleeding on admission | 11 (20.75) | 20 (19.6) | 1 |
| Trauma | 12 (22.64) | 14 (13.7) | 0.18 |
| Transfusion before inclusion* | 29 (54.7) | 56 (54.9) | 0.98 |
| SAPS II | 41 (29 ; 51) | 41 (28 ; 52) | 0.8 |
| SOFA | 6 (4 ; 9) | 7 (4 ; 10) | 0.73 |
| Hb (g/dL) | 11.3±2.5 | 11.3±2.9 | 0.71 |
|  |  |  |  |
| *Organ support during ICU Stay* |  |  |  |
| ICU LOS (Days) | 13 (7 to 22) | 12 (7 to 20) |  |
| Mechanical ventilation | 40 (75.5) | 85 (83.3) | 0.24 |
| Duration of MV (days) | 5 (2 ; 14) | 3 (2 ; 9) | 0.18 |
| Renal support | 7 (13.2) | 9 (8.8) | 0.39 |
| Duration of support (days) | 4 (3 ; 13) | 4 (3 ; 9) | 0.73 |
| Catecholamine | 30 (56.6) | 72 (70.6) | 0.08 |
| Duration of catecholamine (days) | 4 (2 ; 6) | 3 (2 ; 4) | 0.15 |
| *ICU Discharge Blood Tests* |  |  |  |
| Hb (g/dL) | 9.9±1.5 | 9.9±1.2 | 0.88 |
| CRP (mg/L) | 16.05 (3.75 ; 105.7) | 39.9 (4.5 ; 214.9) | 0.32 |
| Ferritin (µg/L) | 554 (327 ; 968) | 380 (174 ; 685) | 0.03 |
| TSAT (%) | 14.5 (11.5 ; 27) | 13 (11 ; 17) | 0.42 |
| Hepcidin (µg/L) | 18.5 (7.9 ; 23.6) | 15.6 (8 ; 27.9) | 0.81 |
| Absolute ID (n) | 34 (64.2) | 60 (58.8) | 0.52 |
| Functional ID (n) | 19 (35.8) | 42 (41.2) |  |

**Table S4 per protocol analysis of primary and secondary outcomes**

In this analysis, we compared the 47 patients with ID treated according to protocol in the intervention arm (excluding the 6 patients with functional ID who received iron alone, without erythropoietin) to the 102 patients with ID not treated in the control arm.

ID, iron deficiency;

|  | n | Patients with ID treated in intervention arm according to study protocol  (n= 47) | n | Patients with ID not treated in control arm  (n=102) | *p* |
| --- | --- | --- | --- | --- | --- |
| Post ICU LOS (Days) | 47 | 49 (15 ; 90) | 102 | 29 (11 ; 90) | *0.44* |
| Number of days alive at home at Day 90 | 47 | 42 (0 ; 76) | 102 | 61 (0 ; 82) | *0.22* |
| Death at Day 90 | 47 | 2 (4.3) | 102 | 17 (16.7) | *0.03* |
| Fatigue (scale 1-10) | 33 | 5.0 (3.0 ; 6.0) | 61 | 6.0 (3.0 ; 7.0) | *0.28* |
| MFI-20 |  |  |  |  |  |
| General fatigue (score 9-45) | 34 | 28 (21 ; 30) | 63 | 26 (18 ; 30) | *0.69* |
| Mental Fatigue (score 6-30) | 34 | 24 (18 ; 27) | 63 | 25 (20 ; 28) | *0.27* |
| Reduced Activity (score 3-15 ) | 34 | 7 (6 ;9) | 63 | 8 (6 ;11) | *0.25* |
| Reduced Motivation (score 2-10) | 34 | 8.(6 ;10) | 63 | 8 (6 ; 10) | *0.64* |
| Day 15 Hb (g/dL) | 33 | 10.9 ±1.5 | 59 | 10.4±1.4 | *0.07* |
| Day 15 Hepcidin (µg/L) | 11 | 34.5 (23.0 ; 53.7) | 31 | 18.0 (7.6 ; 44.0) | *0.07* |

Figure S1: Kaplan-Meier survival curves (till D90)

in patients with hepcidin <41 µg/L treated in the intervention arm according to the study protocol and not treated in the control arm.


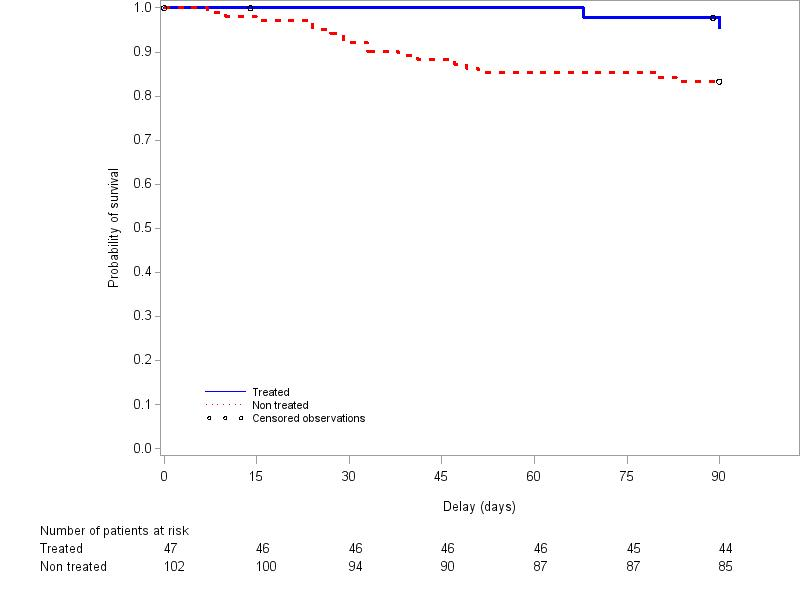


P-value (log-rank) = 0.0369
